# Cell-specific extracellular vesicle-encapsulated exogenous GABA controls seizures in epilepsy

**DOI:** 10.1186/s13287-024-03721-4

**Published:** 2024-04-19

**Authors:** Abhijna Ballal R, Shivakumar Reddy K, Divya Chandran, Sumukha Hegde, Raghavendra Upadhya, Praveen Kumar SE, Smita Shenoy, Vasudha Devi, Dinesh Upadhya

**Affiliations:** 1 Centre for Molecular Neurosciences, Kasturba Medical College, Manipal, Manipal Academy of Higher Education, Manipal, 576104 Karnataka India; 2 Manipal Centre for Biotherapeutics Research, Manipal Academy of Higher Education, Manipal, 576104 Karnataka India; 3 Department of Pharmacology, Manipal Tata Medical College, Jamshedpur, Manipal Academy of Higher Education, Manipal, Karnataka India; 4 Department of Pharmacology, Kasturba Medical College, Manipal, Manipal Academy of Higher Education, Manipal, 576104 Karnataka India

**Keywords:** Extracellular vesicles, Pluripotent stem cells, Neurotransmitter GABA, Epilepsy, Intranasal administration

## Abstract

**Background:**

Epilepsy affects ∼60 million people worldwide. Most antiseizure medications in the market act on voltage-gated sodium or calcium channels, indirectly modulating neurotransmitter GABA or glutamate levels or multiple targets. Earlier studies made significant efforts to directly deliver GABA into the brain with varied success. Herein, we have hypothesized to directly deliver exogenous GABA to the brain with epilepsy through extracellular vesicles (EVs) from human GABA-producing cells and their progenitors as EVs largely mimic their parent cell composition.

**Methods:**

Human neural stem cells (NSCs), medial ganglionic eminence (MGE) cells, and GABAergic interneurons (INs) were generated from induced pluripotent stem cells (iPSCs) and characterized. EVs were isolated from NSCs, MGE cells, and INs and characterized for size and distribution, morphological features, and molecular markers. Exogenous GABA was passively loaded to the isolated EVs as a zwitterion at physiological pH, and the encapsulated dose of GABA was quantified. Epilepsy was developed through status epilepticus induction in Fisher rats by administration of repeated low doses of kainic acid. The extent of the seizures was measured for 10 h/ day for 3–6 months by video recording and its evaluation for stage III, IV and V seizures as per Racine scale. EVs from INs, MGE cells, and NSCs encapsulated with exogenous GABA were sequentially tested in the 4^th^, 5^th,^ and 6^th^ months by intranasal administration in the rats with epilepsy for detailed seizure, behavioral and synapse analysis. In separate experiments, several controls including exogenic GABA alone and EVs from INs and MGE cells were evaluated for seizure-controlling ability.

**Results:**

Exogenic GABA could enter the brain through EVs. Treatment with EVs from INs and MGE cells encapsulated with GABA significantly reduced total seizures, stage V seizures, and total time spent in seizure activity. EVs from NSCs encapsulated with GABA demonstrated limited seizure control. Exogenic GABA alone and EVs from INs and MGE cells individually failed to control seizures. Further, exogenic GABA with EVs from MGE cells improved depressive behavior while partially improving memory functions. Co-localization studies confirmed exogenous GABA with presynaptic vesicles in the hippocampus, indicating the interaction of exogenous GABA in the brain with epilepsy.

**Conclusion:**

For the first time, the study demonstrated that exogenous GABA could be delivered to the brain through brain cell-derived EVs, which could regulate seizures in temporal lobe epilepsy. It is identified that the cellular origin of EVs plays a vital role in seizure control with exogenous GABA.

**Supplementary Information:**

The online version contains supplementary material available at 10.1186/s13287-024-03721-4.

## Introduction

Epilepsy affects ∼60 million people worldwide. Nearly 30% of patients with epilepsy have temporal lobe epilepsy (TLE), characterized by the progressive development of complex partial seizures and hippocampal neurodegeneration associated with co-morbidities such as cognitive and mood impairments [1, 2]. While antiseizure medications (ASMs) have been valuable for seizure control in most patients, ∼30–40% of patients typically develop pharmaco-resistant or intractable epilepsy, defined as failure of two ASMs given at apt doses [3, 4]. Although 15 new compounds have been introduced in the last 25 years, the overall proportion of patients with refractory epilepsy remains unchanged [4, 5]. Most of the ASMs in the market are voltage-gated sodium channel blockers that reduce the action potential generation and limit neuronal firing [6]. Apart from sodium channel blockers, other medications target voltage-gated calcium or potassium channels or modulate GABA or glutamate levels or presynaptic activity, while some medications act on multiple targets [7]. Also, most of these ASMs are associated with various side effects [8–9).

Hyperactivity of excitatory glutamatergic neurons or reduced activity of inhibitory GABAergic interneurons that cause a persistent imbalance in the excitatory-inhibitory neurotransmission could trigger seizures [10–13]. Thus, a treatment that directly reduces the activity of excitatory neurons or increases the inhibitory neurotransmission can potentially control seizures [14, 15]. Multiple studies demonstrated the enhancement of endogenous GABA signalling through transplanted GABAergic interneuron progenitors, resulting in seizure control [15–20]. Also, based on the preclinical efficacy of GABA-secreting interneuron control of seizures, the first human Phase I/II clinical trial (NCT05135091) on inhibitory interneurons in drug-resistant unilateral mesial temporal lobe epilepsy is in progress. In this study, one group of subjects are being treated with single stereotactic intracerebral administration of GABA-secreting interneurons, compared to the other group, which received sham treatment. The subjects are being assessed for safety, tolerability, neural cell viability, local inflammation, and seizure frequency reduction from baseline over a two-year post-treatment period and followed up for 13 more years. Promising results from two recruited patients were updated at the American Academy of Neurology (AAN) 2023 annual meeting, suggesting that direct modulation of GABA or glutamate levels could be an ideal strategy for treating epilepsy.

To directly supply GABA to enhance its concentration in the brain, it should cross the blood-brain barrier (BBB). However, no human data is available to demonstrate BBB permeability to GABA [21]. Studies in the 1960s used direct GABA to control seizures with limited success, possibly due to the impermeable BBB [22]. Animal models have demonstrated the impermeability of GABA to the brain [23–24]. Thus, an efficient delivery system is required to deliver GABA directly to the brain for its functional effects. Earlier studies made significant efforts to deliver GABA to the brain with varied success (25, 26). Extracellular vesicles (EVs) with nano size and their recently established potential for drug delivery make them the ideal choice as they could reach deeper parts of the brain upon intranasal delivery [27–28]. As EVs mostly mimic their parent cell composition, it is ideal to utilize brain cell derived EVs, particularly for brain delivery. As GABA is produced from GABAergic interneurons, interneurons or their progenitor cell derived EVs are the best bet to carry GABA to the brain with epilepsy. Also, exogenous GABA could be passively loaded into the EVs as a small hydrophilic molecule and zwitterion at physiological pH. In this aspect, we have tested the hypothesis of direct exogenous GABA delivery to the brain through cell-specific EVs to evaluate their seizure-controlling capability in a kainate-induced rat model of temporal lobe epilepsy.

## Materials and methods

**Fig. 1:**
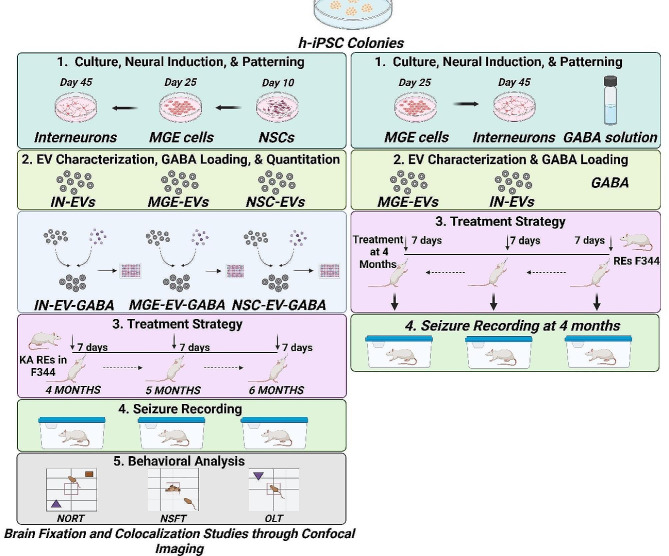
Experimental plan. (1) Human iPSCs were induced to generate neural stem cells (NSCs) and patterned to develop medial ganglionic eminence cells (MGE) and GABAergic interneurons (IN). These cells were molecularly characterized for stages of development. (2) EVs from NSCs, MGE cells and interneurons were isolated, characterized and loaded with GABA using different methods. The amount of EV encapsulated GABA from each source and method was quantified using ELISA. 3 and 4. Rats with epilepsy (REs) were generated using the Kainic acid model in F344 rats (*n* = 32), and seizures were scored continuously from 3–6 months with video recording. At 4 months, REs were intranasally treated with interneuron-derived EV-encapsulated GABA (IN-EV-GABA) daily for 7 days. At 5 months, the same rats were treated with MGE-derived EV encapsulated GABA (MGE-EV-GABA) daily for 7 days, and at 6 months, the same rats were treated with NSC-derived EV-encapsulated GABA (NSC-EV-GABA) daily for 7 days. In another set of experiments, MGE-EVs, IN-EVs and GABA were intranasally administered daily for a week to REs separately at 4 months, and seizures were recorded. 5. Another batch of REs was treated with MGE-EV-GABA for 7 days and tested for detailed behavioral analysis before and during the treatment. GABA was conjugated with o-phthaldialdehyde (OPA), and EV-encapsulated GABA-OPA was intranasally administered and traced in the brain. Following brain isolation and fixation, immunostaining for presynaptic marker synaptophysin was performed, and 0.7 μm confocal Z-sections were evaluated at 630 magnifications

The schematic experimental design is provided in Fig. 1.

### Generation of neural stem cells, medial ganglionic eminence cells, and interneurons

Human induced pluripotent stem cells ND 1.4 (RRID: CVCL_1E77, (NHCDR Cat# ND50021, P31) were procured from the National Centre for Cell Science in Pune, India. These lines were produced from episomal reprogramming of healthy male neonatal fibroblasts. Cells were thawed and cultured on a matrigel with TeSR E8 medium. Once cells attained 70% confluency, cells were detached with dispase solution and allowed to develop embryoid bodies (EBs). On day 4, neural lineage was induced with a neural induction medium (NIM) containing DMEM/F12, nonessential amino acid solution (NEAA), N2 supplement, and heparin. These treatments continued until day 7. On day 7, EBs were attached to culture plates coated with laminin and continued until day 10 to form neural rosettes containing neural stem cells (NSCs). On day 10, NIM was added with sonic hedgehog to pattern forebrain progenitors. On day 16, neural rosettes were detached, and NIM containing B27 without retinoic acid was added to form neurospheres. Neurospheres were grown until day 25 to form medial ganglionic eminence (MGE) cells. Single cells and 4–6 cell clumps were prepared from MGE cells and cultured on poly-L ornithine and laminin-coated multiple 6-well plates until 45 days with neural differentiation medium containing neurobasal medium, NEAA, N2 and B27 supplements along with IGF-1, cAMP, BDNF and GDNF. The identity of NSCs was confirmed with SOX 2 and NESTIN co-immunostaining, while MGE cells were confirmed with NKX2.1 immunostaining and interneurons with β-III tubulin and GABA co-immunostaining.

### Isolation of EVs from neural stem cells, medial ganglionic eminence cells, and interneurons

Extracellular vesicles were isolated from NSCs, MGE cells, and interneurons by collecting media supernatants on days 10, 25, and 45, respectively. Cellular debris in the media was removed by centrifugation at 2000 g for 30 min, and EVs were isolated using the established methods [29–33]. Briefly, an equal amount of 2x of 12% polyethylene glycol (w/v, 6000, Sigma-Aldrich 81,260) in 1 M NaCl was added to the media, mixed thoroughly, and kept for 14 h at 4 °C. The solution was centrifuged in a tabletop centrifuge at a speed of 3300xg at 4 °C for one hour. Following high percentage polyethylene glycol isolation, the supernatant was discarded, and the pellet was suspended with 3 ml of PBS and an equal amount of 2x of 5% polyethylene glycol, incubated for an hour at 4 °C and centrifuged again at a speed of 3300xg at 4 °C for one hr. Following final centrifugation in this dual precipitation method, the supernatant was discarded, and the pellet was suspended in 200 µl of PBS and used for various experiments.

### Characterization of isolated EVs

Isolated EVs from NSCs, MGE cells and INs were characterized for size distribution, ultrastructure, and molecular markers [34]. The average size and concentration of isolated EVs were determined by nanoparticle tracking analysis using Nanosight LM10 (Malvern Instruments, UK). Samples were vortexed to avoid clumps and diluted up to 1:10000 with sterile PBS to achieve consistent particle concentration within the optimal detection range. The ultrastructure of the isolated EVs was evaluated using transmission electron microscopy. Western blotting was performed for TSG 101 (rabbit polyclonal antibody from Invitrogen, Cat # PA5-31260) CD 63 (rabbit polyclonal antibody from Sigma Aldrich, Cat # SAB4301607), GRP 94 (rabbit polyclonal antibody from Sigma Aldrich, Cat # SAB2101094) and β-actin (mouse monoclonal antibody from Invitrogen, Cat # MA1-140). All the culturing, isolation, and detailed characterization for EVs from individual cell types were performed in triplicates. Results of the size and concentration of EVs were expressed as Mean ± standard error of the mean (SEM).

### Encapsulation of GABA to extracellular vesicles and its estimation

Since we planned to use EVs from different sources, simple passive loading was used to encapsulate GABA at room temperature (RT) for one hour [35]. Many trials with varying concentrations of GABA (500 µg/ml, 1 mg/ml, 5 mg/ml, 10 mg/ml, etc.) were used to load GABA to EVs in PBS (pH-7.2) to identify the highest amount of GABA encapsulation in EVs. Following GABA loading into EVs, to remove the non-loaded GABA, the solution was incubated with an equal amount of 2 X of 5% polyethylene glycol, set for an hour at 4 °C and centrifuged at a speed of 3300 x g at 4 °C for one hour. The supernatant was discarded following centrifugation, and the pellet was suspended in 200 µl of PBS. The total GABA concentration in EVs was estimated using an ELISA (Cat# BA-E-2500, ImmuSmol, France) as per the manufacturer’s instructions.

### Derivatization of GABA with OPA

To identify GABA inside the rat brain, GABA was conjugated with o-phthaldialdehyde (OPA) and its passive loading into the EVs for intranasal delivery. GABA was conjugated with OPA using 0.1 M borate buffer, mercaptopropionic acid, and OPA reagent. The Econo chromatography column (cat.no 7,371,522, 1.5 × 20 cm, Bio-Rad) was cast with Sephadex G-10 gel filtration resin. It was prepared by adding 5 g of Sephadex G-10 to 12 ml of 10 mM PBS with gentle and thorough mixing. The upper aqueous layer was aspirated from the mixture with fresh 10 mM PBS and left to stand to facilitate sedimentation overnight. Then, the concentrated solution was loaded into the column. The GABA with OPA was injected into the column, eluting at a flow rate of 0.20 ml/min with 10 mM PBS. About 15–20 ml of eluent was collected in each tube using an auto-collector. After 40 min of running, fractions containing GABA (Figure S1) were collected in tubes covered with foil to protect them from light. During this peak, the eluent is collected and concentrated at 200 µg. Eluents were pooled and concentrated with protein filters to get the desired concentration of conjugated GABA.

### Tracking of GABA loaded EVs in naïve rat brain

For tracking GABA-loaded EVs and to ensure they reached the different regions in the rat brain, we have derivatized GABA with OPA, and EVs were labelled with red cell linker dye PKH26 (27). The brief procedure is as follows. The naïve rats (non-epileptic, *n* = 4) were anesthetized by intraperitoneal injection of ketamine + xylazine cocktail and intranasally administered with EVs encapsulated with OPA-conjugated GABA. Rats were held ventral-side up, and slowly using a 10 µl micropipette administration was done into either nare at 5–10 µl increments at 5-minute intervals for 30 µl /each rat, containing EVs equivalent to 100 µg EV protein. After 6 h, the rats were sacrificed and perfused with normal saline and 4% PFA. The brain was shelled out and collected in a tube containing 4% PFA and kept overnight, then the solution was changed to PB and kept for 24 h. After 24 h, the solution was changed to a graded sucrose solution of 15% and 30% sucrose until the brain sinks. Later, the 10 μm sectioning was taken on gelatin-coated slides using a cryostat. Finally, sections were washed with PBS, counterstained with DAPI and fluorescence images were taken. A representative image is provided in Supplementary Figure S3.

### Development of the epilepsy model

Following Kasturba Medical College Institutional Animal Ethics Committee approval (IAEC/KMC/39/2018) for animal experiments, Fisher 344 rats were procured from the National Institute of Nutrition, Hyderabad, and acclimatized for two weeks before any procedure. They were provided with ad-libitum food and water, and a 12 h day/night cycle was maintained. Six to eight weeks-old rats were taken for the experiment. The rats were weighed, and kainic acid (Cayman Pharma, United States) doses were calculated according to weight.

Status epilepticus (SE) was induced in 7–9 rats in each batch. Intraperitoneal (i.p) kainic acid at 5 mg/kg concentration was given hourly for 3–5 h to induce status epilepticus. The Racine scale was considered to identify different seizure stages during the procedure. When the rat showed first stage III (unilateral forelimb clonus) or stage IV characterized by bilateral forelimb clonus and rearing or first stage V seizure displaying bilateral forelimb clonus with rearing and falling, the time was noted down and observed for continuous stage III-V seizure for 2 h. After 2 h of status epilepticus, the rats were given an i.p injection of 10 mg/kg of diazepam [36].

### Scoring of spontaneous recurrent motor seizures

The appearance of Spontaneous recurrent motor seizures (seizures) after the latent period is the beginning of the chronic phase. The rats were kept in a transparent cage with proper bedding and provided ad libitum access to food and water. The extent of the seizures was measured for 10 h/day. From the beginning of 3rd month to the end of 6 months, seizures were continuously monitored through video recordings. The frequency of stages III, IV, and V seizures was noted, and each seizure’s duration was noted. The average number and duration of each seizure for the individual animal throughout the period were measured.

### Treatment of epilepsy with GABA-loaded EVs

For identifying the in vivo efficacy, 32 rats were used for the study. Rats with epilepsy (REs, *n* = 24) were generated using the kainic acid model in F344 rats. In the 3rd month, we started recording seizures (and continued up to 6 months). For up to 4 months, no treatments were given to nullify the impact of evolving seizures over the period. At 4 months, rats with epilepsy (*n* = 8) were intranasally treated with interneuron-derived EV encapsulated with GABA (IN-EV-GABA) daily for 7 days. For the remaining period until 5 months, these rats were kept without treatment as a washout period for the IN-EV-GABA. At 5 months, the same rats (*n* = 8) were treated with MGE-derived EV encapsulated with GABA (MGE-EV-GABA) daily for 7 days. The remaining period until 6 months, these rats were kept without treatment as a washout period for the MGE-EV-GABA. At 6 months, the same rats (*n* = 8) were treated with NSC-derived EV encapsulated with GABA (NSC-EV-GABA) daily for 7 days. Thus, the same rats were used for all these treatments. We started with EVs produced from interneurons (IN-EV-GABA), which are GABA-producing interneurons. Since we found this treatment was effective, we used EVs produced from interneuron precursor cells (MGE-EV-GABA) in the next step, as they are easier to make. Since we found this treatment also effective, we used EVs from further earlier lineage cells, i.e., neural stem cells (NSC-EV-GABA).

Another batch of REs (*n* = 8) was treated with MGE-EV-GABA for 7 days and tested for detailed behavioral analysis before and during the treatment, along with naïve rats (*n* = 8) for comparison. Another group of REs (*n* = 8) were used to evaluate the effect of exogenic GABA alone or IN-EVs or MGE-EVs in controlling seizures.

Rats were anaesthetized with a ketamine + xylazine cocktail. GABA-loaded EVs were administered intranasally at 30 µl for each nostril slowly and carefully without damaging the mucous membrane with 10ul spurts. All the rats were video recorded and analyzed daily 10 h for 7 days to evaluate total behavioral seizures, stages III, IV, and V seizures, based on the Racine scale. Also, total time spent in seizures /10 h, time spent in stage III seizures /10 h, time spent in stage IV seizures /10 h, and time spent in stage V seizures /10 h frequency were analyzed.

### Behavioral tests to examine memory and mood functions

Epilepsy is associated with memory deficits and depression in rats. The following tests were performed for memory function: (1) Object location test (OLT), (2) Novel object recognition test [37]. The object-based tests (NORT and OLT) were performed using an open-field apparatus measuring 100 cm x 100 cm. A novelty-suppressed feeding test (NSFT) was performed to evaluate depressive-like behavior in these rats.

### Novel object recognition test (NORT)

NORT is used to check the competence of animals for recognition memory. A rat was observed in an open field with three successive trials separated by 15-minute intervals. In brief, each rat was placed in an open field for 5 min in the first trial for acclimatization to the testing apparatus (habituation phase), whereas, in trial 2, the rat was allowed to explore two identical objects placed in distant areas of the open field (sample phase). In trial 3 (testing phase), one object was kept the same (Known object), and another object was replaced by a new object (novel object). Trials 2 and 3 were video recorded to measure the number of visits to each object and the amount of time spent with each of the two objects. Rats with normal brain function spent much of their exploration time with a novel object than the known object.

### Object location test (OLT)

This test examines the cognitive ability to detect subtle changes in the immediate environment based on the rodent’s innate preference for novelty. A rat was observed in an open field with three successive trials separated by 15-minute intervals. In brief, each rat was placed in an open field for 5 min in the first trial for acclimatization to the testing apparatus (habituation phase), whereas, in trial 2, the rat was allowed to explore two identical objects placed in distant areas of the open field (sample phase). In trial 3 (testing phase), one of the objects was moved to a new location (novel place) while the other object remained in the last place (familiar place). Trials 2 and 3 were video-recorded to measure the time spent with the two objects. The results were computed, such as the percentage of time spent exploring the objects in a familiar place and novel place and the total object exploration time in trial 3. Rats with normal hippocampal function could recognize minor changes in the immediate environment and tend to spend more time with the object in a novel place. However, object exploration time changes with the level of hippocampal impairment.

### Novelty-suppressed feeding test (NSFT)

NSFT assesses the ability of the animal to resolve a conflict between a context that induces heightened anxiety and a drive to approach an appetitive stimulus. This test comprised two days of monitoring and examining the extent of depressive-like behavior in animals using a home cage box. Following 24 h of food deprivation, regular food was placed on a small white paper in one corner of the box (this was performed on a home cage box to reduce anxiety), and each rat was placed diagonally opposite to the position of the food in the novel open field box. The time each rat reached the food and the first bite was recorded in all the groups. The start of eating was considered when the rat bites the first pellets using its forepaws for the first time. For the rat that failed to eat food in the 5-minute duration of this test, the latency value was 300 s. Once the test was done, the rat was given adequate access to food and water.

### Tracking of exogenous GABA in the brain

Rats (*n* = 4) were intranasally administered with EVs encapsulated with OPA-conjugated GABA to track the exogenously loaded GABA in the brain with epilepsy. After 6 h of intranasal administration, rats were sacrificed and perfused with normal saline and 4% PFA. The brain was shelled out and collected in a tube containing 4% PFA and kept overnight; then, the solution was changed to PB and kept for 24 h. After 24 h, the solution was changed to a graded sucrose solution of 15% and 30% sucrose until the brain sank. Later, 10 μm sections were taken on gelatin-coated slides by using a cryostat. Following blocking with donkey serum, the anti-synaptophysin antibody was added and incubated at 4 °C overnight, followed by a secondary antibody for 1 h. Slides were washed, counterstained with DAPI containing antifade, and scanned under a Leica SP5 confocal microscope. Z sections of 0.7 μm at 630 magnification were taken at different hippocampus regions.

#### Statistical analysis

All the data were analyzed using GraphPad Prism software and expressed as mean ± SEM. A one-way analysis of variance was used to find the significance of the study groups in all the experiments except for NORT and OLT behavioral analysis, where an unpaired t-test was used. *p* < 0.05 was considered statistically significant.

## Results

### Confirmation of neural stem cells, medial ganglionic eminence cells and interneurons

**Fig. 2:**
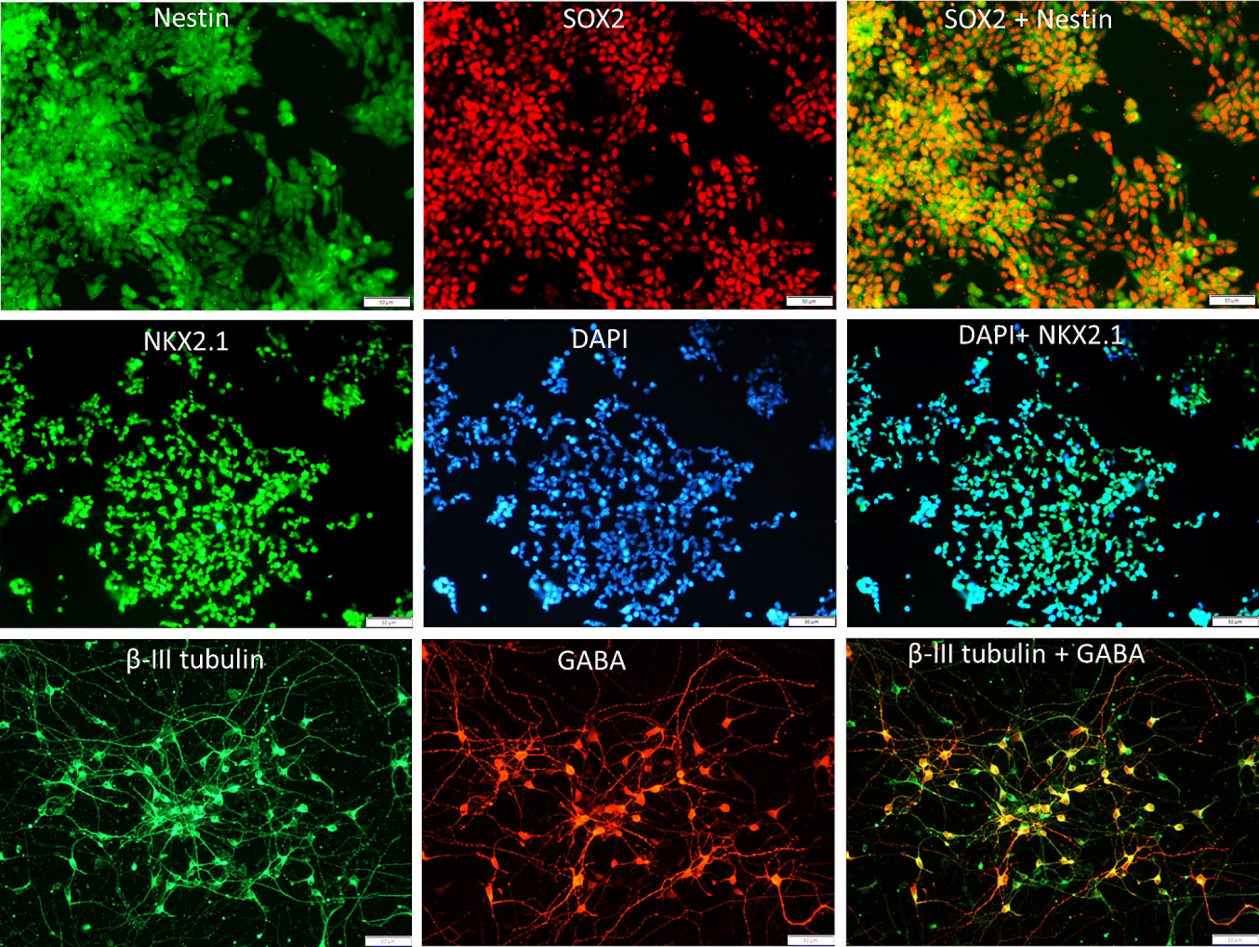
Generation of NSCs, MGE and interneurons from human-induced pluripotent stem cells. The top panel represents neural stem cells on day 10 positive for NESTIN (green) and SOX 2 (red), and the merged image demonstrates cells positive for both NESTIN and SOX2 (orange). The middle panel represents medial ganglionic eminence (MGE) cells on day 25 positive for NKX2.1 (green) and DAPI (blue), and the merged image demonstrates cells positive for NKX2.1 and DAPI. The lower panel represents GABAergic interneurons on day 45, positive for β-III tubulin (green) and GABA (red), and the merged image demonstrates cells positive for both β-III tubulin and GABA (orange)

Neural stem cells (NSCs), medial ganglionic eminence (MGE) cells and GABAergic interneurons (INs) were generated from human induced pluripotent stem cells through directed differentiation protocols. We confirmed the generation of neural stem cells through Nestin and SOX2 immunostaining (Fig. 2 upper panel) on day 10. The identity of MGE cells was confirmed through immunostaining for NKX2.1 (Fig. 2, middle panel) following single-cell generation from the neurospheres on day 25. Generated GABAergic interneurons were confirmed through the co-immunostaining for β-III tubulin and GABA (Fig. 2, lower panel) on day 45. Differentiation efficiency for NSCs was > 95%, MGE cells was > 92%, and INs was > 90%.

### Isolation of extracellular vesicles from different cell types and their characterization

**Fig. 3:**
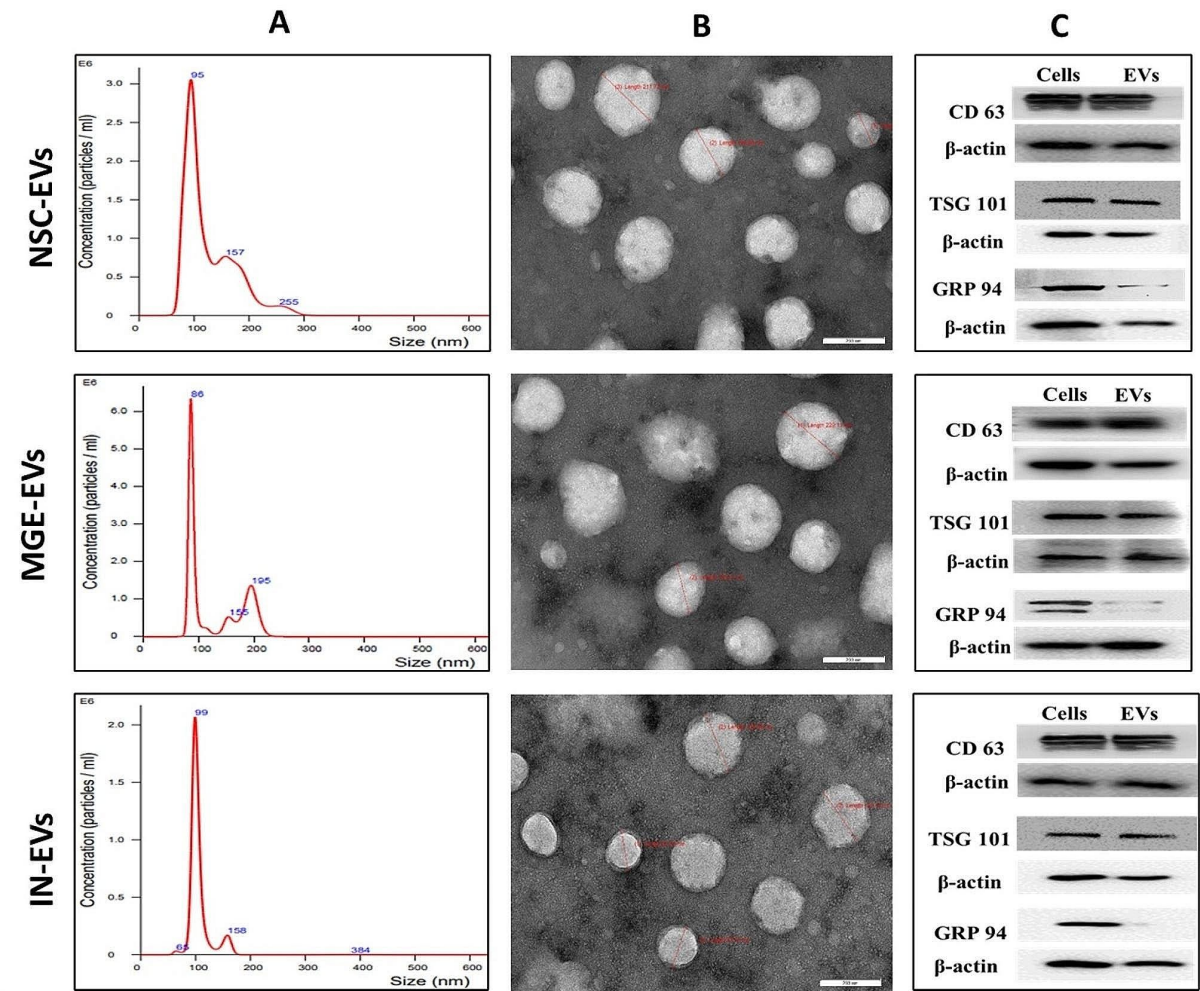
Characterization of isolated extracellular vesicles. Figure 3A represents the size distribution of isolated EVs demonstrated by Nanosight LM10 for neural stem cell-derived EVs (NSC-EVs), medial ganglionic eminence cell-derived EVs (MGE-EVs) and interneuron derived EVs (IN-EVs). Figure 3B represents TEM images of isolated NSC-EVs, MGE-EVs and IN-EVs with a scale bar of 200 nm. Figure 3C demonstrates that NSC-EVs, MGE-EVs and IN-EVs are positive for EV cytoplasmic marker TSG 101 and EV transmembrane marker CD 63. At the same time, they are mostly negative for GRP 94, an endoplasmic reticulum marker. The presence of β-actin is demonstrated for all the markers. Cells are positive references for CD63, TSG101 and GRP94 in all the groups

Extracellular vesicles were isolated from NSCs, MGE cells, and interneurons by collecting media supernatants on days 10, 25, and 45, respectively. Isolated EVs were characterized by size and number using Nanosight LM10 (Fig. 3A). The amount of EVs isolated per ml of media differed between the groups due to different cell culture conditions for different types of cells. However, in all the groups (*n* = 3/group), no significant difference in EV size distribution was observed (Fig. 3A). The mean size of EVs from NSCs, MGE cells and interneurons was 153.7 ± 12.99, 146.9 ± 21.4 nm and 159.7 ± 11.57 nm, respectively, while the mode size was 105.3 ± 6.39, 98.67 ± 13.27 and 103.3 ± 7.54 nm respectively. TEM analysis revealed that most EVs isolated from NSCs, MGE cells and interneurons with a diameter < 200 nm size and comparable morphology (Fig. 3B). Molecular characterization of isolated particles from NSCs, MGE cells and interneurons revealed that they were positive for EV cytoplasmic marker TSG 101 and EV transmembrane marker CD 63. At the same time, they were negative for GRP 94, a marker for non-EV fractions such as endoplasmic reticulum (Fig. 3C and Figure S2). These results confirm that isolated particles from NSCs, MGE cells, and interneurons were enriched in EVs. We consider their size ideal for loading GABA or its agonist to make them therapeutically applicable for treating GABA deficiency disorders such as epilepsy since they could easily pass through the BBB.

### Quantitation of EV-encapsulated GABA

Extracellular vesicle encapsulation of exogenous GABA through co-incubation at room temperature with different concentrations yielded different results. The maximum amount of GABA loaded was 288.5 µg/ml (8.7 µg/30ul for each nostril), containing 100 µg EV protein with a loading efficiency of 9.6%. The number of NSC-EVs, MGE-EVs and IN-EVs in 30ul of nasal administration was 1.96 (± 0.23) X 10^9^, 1.82 (± 0.15) X 10^9^ and 1.94 (± 0.45) X 10^9^ respectively. No difference in loading efficiency was observed for different EV sources.

### Exogenic GABA entered the brain and regulated seizures in rats with epilepsy

Before using the GABA-loaded EVs for treating rats with epilepsy, it is required to ensure they reach the hippocampus following intranasal administration. We have observed OPA and PKH 26 labelled EVs in different rat brain regions (*n* = 4), including the hippocampus. A representative image is provided in Figure S3.

**Fig. 4:**
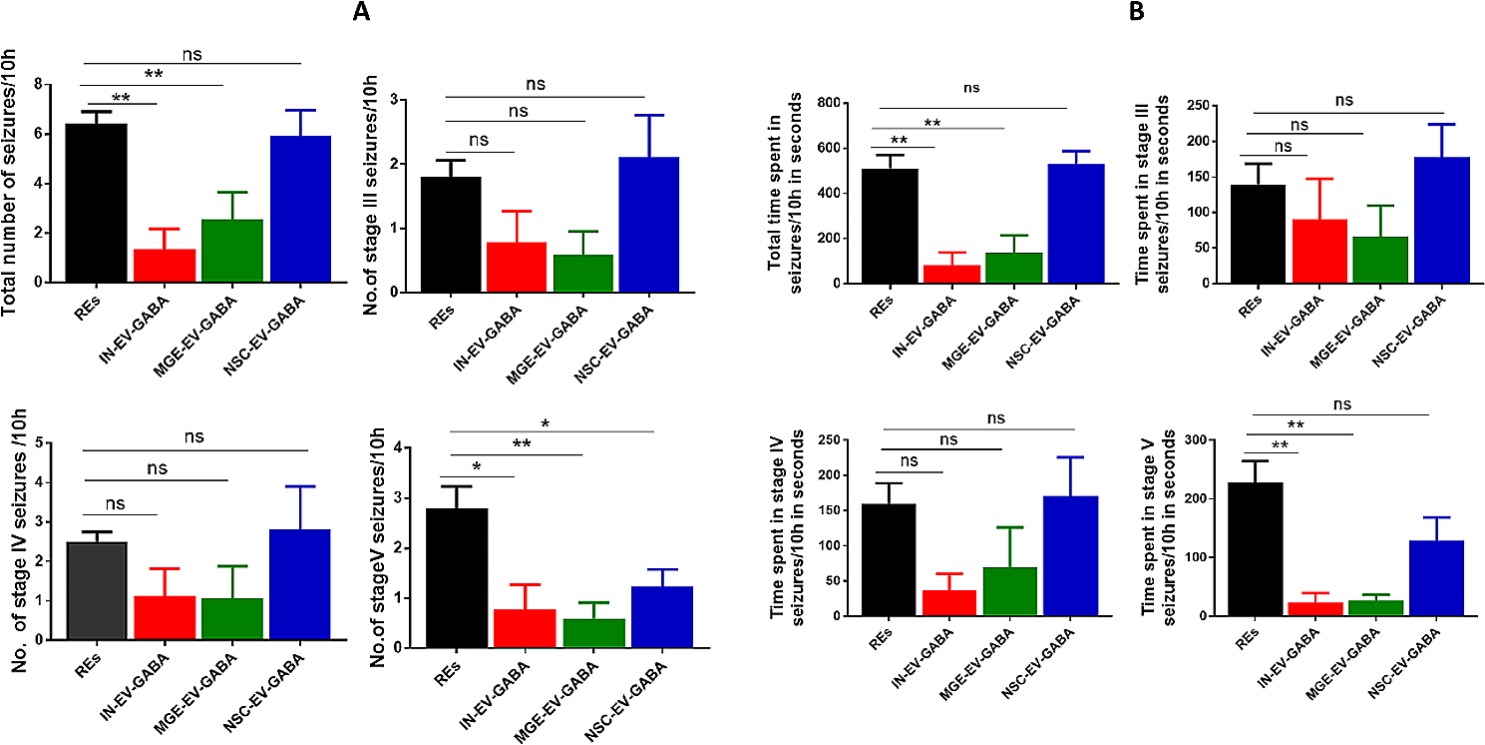
Effect of intranasal administration of GABA-loaded EVs on the number of seizures and time spent in seizures. 4 A: The total number of seizures per 10 h and the number of stage III, IV and V seizures per 10 h in a group of REs were compared to the same rats treated with NSC-EV-GABA, MGE-EV-GABA and IN-EV-GABA. 4B. The total time spent in seizures, time spent in stage III, IV and V seizures per 10 h (seconds) in MGE-EV-GABA, NSC-EV-GABA and IN-EV-GABA groups were compared to a group of untreated REs. REs: Rats with epilepsy; MGE-EV-GABA: GABA loaded into EVs derived from medial ganglionic eminence cells; IN-EV-GABA: GABA loaded into EVs derived from interneurons. For multiple comparisons between the groups, Newman-Keuls post hoc test was used. **p < 0.01; *p < 0.05, ns: non-significant

Administration of kainic acid-induced epilepsy in the rats with stage III, IV and V seizures (Figure S4). Scoring of seizures based on Racine scale demonstrated stage III seizures displaying unilateral forelimb clonus (Figure S4, left panel), stage IV seizures showing bilateral forelimb clonus and rearing (Figure S4, middle panel) and stage V seizures displaying bilateral forelimb clonus with rearing and falling (Figure S4, right panel). Seizures were continuously recorded 10 h/day from 3 to 6 months through video recording. The total number of seizures, stage III, stage IV and stage V seizures were provided in Fig. 4A. The total time spent in seizure activity, stage III, stage IV, and stage V seizures of these REs are provided in Fig. 4B.

Treatment with IN-EV-GABA daily once for a week at 4 months in these REs resulted in a significant decline in the total number of seizures (*p* < 0.01, Fig. 4A), stage V seizures (*p* < 0.05, Fig. 4A), total time spent in seizure activity (*p* < 0.01, Fig. 4B) and time spent in stage V seizure activity (*p* < 0.01, Fig. 4B) compared to pretreatment. However, stage III and IV seizures and the time spent in these seizures were unaltered before and after treatment (Fig. 4A and B).

Treatment with MGE-EV-GABA daily once for a week at 5 months in these REs resulted in a significant decline in the total number of seizures (*p* < 0.01, Fig. 4A) and stage V seizures (*p* < 0.01, Fig. 4A), total time spent in seizure activity (*p* < 0.01, Fig. 4B) and time spent in stage V seizure activity (*p* < 0.01, Fig. 4B) compared to pretreatment period. However, stage III and IV seizures and the time spent in these seizures were unaltered before and after treatment (Fig. 4A and B).

Upon administration of NSC-EV-GABA daily once for a week at 6 months, the treatment failed to reduce the total number of seizures (*p* > 0.05, Fig. 4A) and stage V seizures (*p* > 0.05, Fig. 4A), total time spent in seizure activity (*p* > 0.01, Fig. 4B) in the REs compared to pretreatment period. However, time spent in stage V seizure activity was reduced (*p* < 0.01, Fig. 4B) with NSC-EV-GABA treatment compared to the pretreatment period. Also, stage III and stage IV seizures and the time spent in these seizures were unaltered before and after treatment in the REs (Fig. 4A and B).

**Fig. 5:**
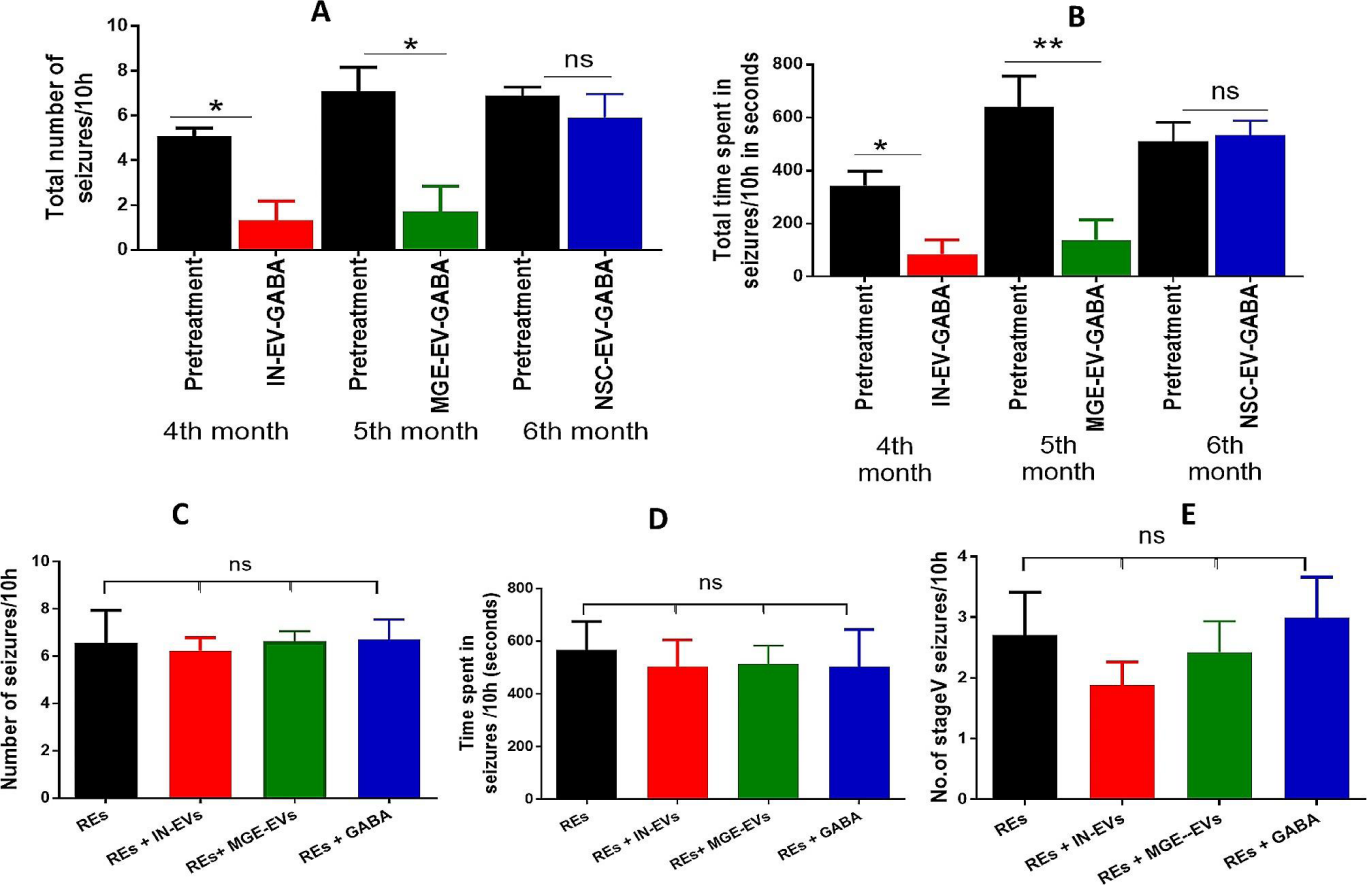
Seizure data analysis at different times and groups. Analysis of individual pretreatment period seizure data with treatment data using ANOVA identified a significant difference in the total number of seizures (A) and total time spent in seizure activity (B) in the MGE-EV treatment group. In the IN-EV-GABA group, the total number of seizures was significantly reduced compared to its pretreatment period, while time spent in seizure activity was non-significantly lower. However, NSC-EV-GABA treatment didn’t demonstrate any significance compared to its pretreatment period. C: Effect of intranasal administration of IN-EVs, MGE-EVs and GABA alone as 3 different controls on total number of seizures in REs. D. Effect of intranasal administration of IN-EVs, MGE-EVs and GABA alone on the number of stage V seizures in REs. E. Effect of intranasal administration of IN-EVs, MGE-EVs and GABA alone on the total time spent in seizure per 10 h in REs. REs: Rats with epilepsy. For multiple comparisons between the groups, Newman-Keuls post hoc test was used. **p < 0.01; *p < 0.05. In Figure B, *p < 0.05 when analysed with an unpaired t-test between the pretreatment period and the treatment period, but not with Newman-Keuls post hoc test. ns: non-significant

Evaluation of seizures among IN-EV-GABA, MGE-EV-GABA, and NSC-EV-GABA groups identified no significant difference in the total number of seizures (*p* > 0.05, Fig. 5A) and stage V seizures (*p* > 0.05, Fig. 5B) among the treatment groups. Additional analysis of individual pretreatment period seizure data with treatment data (Fig. 5A&B) using ANOVA with Newman-Keuls post hoc test identified a significant difference in the total number of seizures (5A) in the MGE-EV-GABA treatment group and IN-EV-GABA groups. In contrast, the total time spent in seizure activity (5B) was reduced only in the MGE-EV-GABA group with the post hoc test. If an unpaired t-test between the pretreatment and treatment period of the IN-EV-GABA group was performed, a significant reduction in the total time spent was demonstrated with the IN-EV-GABA treatment. However, treatment with NSC-EV-GABA failed to demonstrate any significance compared to its pretreatment period.

### Exogenic GABA alone or IN-EVs or MGE-EVs failed to control seizures

Since exogenic GABA demonstrated seizure-controlling ability when encapsulated in IN-EVs or MGE-EVs, we tested with multiple controls, such as exogenic GABA alone or MGE-EVs, and IN-EVs alone could control seizures (Fig. 5C-E). For this, GABA was intranasally administered at 8.7 µg/30ul of PBS for each nostril and 30 µl of EVs in PBS from interneurons and MGE cells equivalent to 100 µg EV proteins, each treatment for a week in REs. No significant reduction in the total number of seizures (5C), stage V seizures (5D) or time spent in seizure activity (5E) was observed in GABA alone, IN-EVs or MGE EVs group compared to untreated REs (Fig. 5C-E).

Overall, seizure data demonstrated exogenic GABA or cell specific EVs alone failed to control seizures. However, IN-EV-GABA and MGE-EV-GABA treatments significantly reduced seizures, while NSC-EV GABA showed minimal seizure control. These data highlight that exogenic GABA could enter the brain and, with cargo specific EVs, control seizures in temporal lobe epilepsy.

### Behavioral analysis reveals partial improvement in cognitive functions

Since IN-EV-GABA and MGE-EV-GABA treatment demonstrated a comparable extent of seizure control, as MGE cells are easy to produce compared to interneurons, we have performed detailed behavioral studies only with the MGE-EV-GABA treatment group, and the results were compared to the pretreatment REs and naïve rats.

### MGE-EV-GABA improves novel object recognition memory

**Fig. 6:**
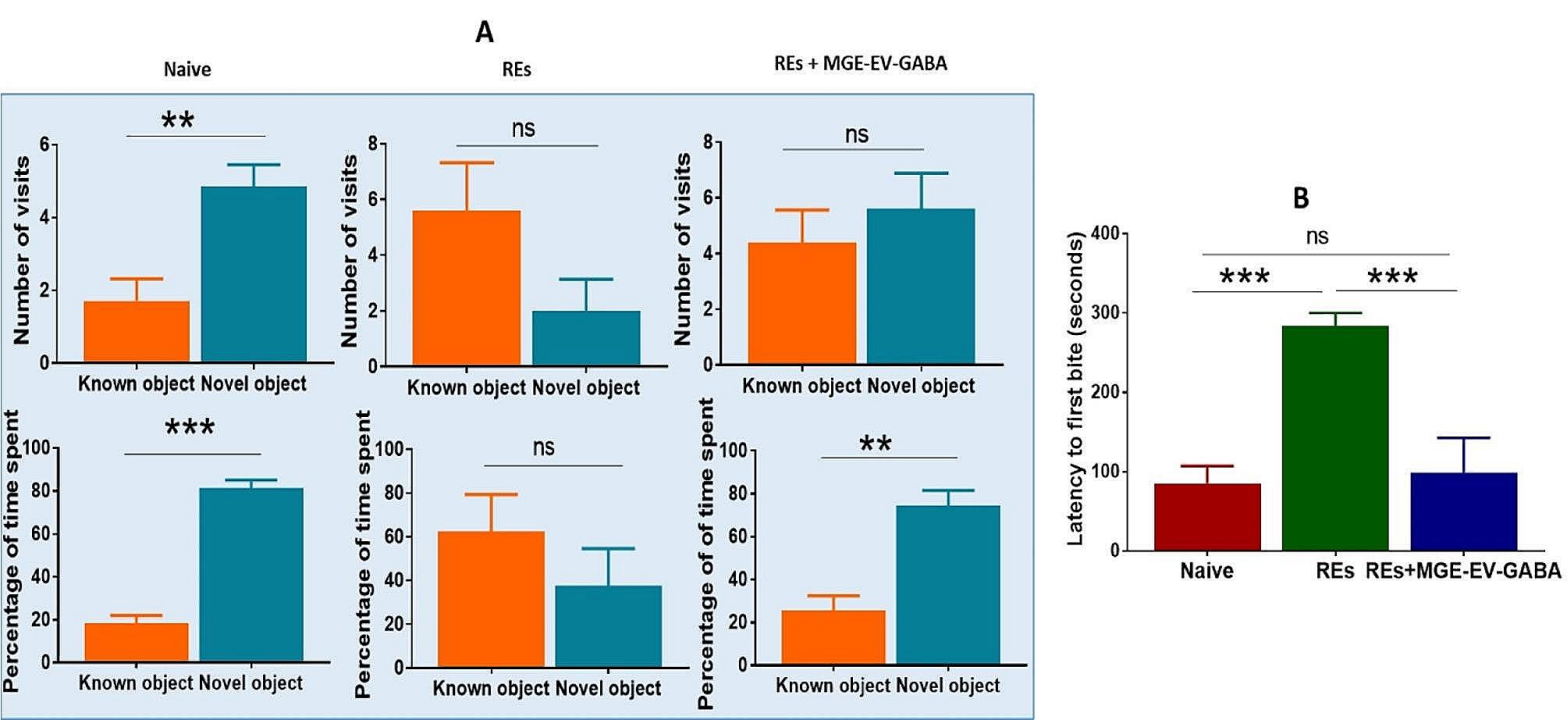
Effect of intranasal administration of GABA-loaded EVs on a novel object recognition test (A) and novelty suppressed feeding test (B). 6 A: The upper panel demonstrates the number of visits to novel and known objects in trial 3 from the group belonging to naïve rats, REs and REs treated with MGE-EV-GABA. The lower panel demonstrates the total time spent with each object in trial 3 from the group of naïve rats, REs and REs treated with MGE-EV-GABA. 6B: This test demonstrates latency to first bite in rats upon 24 h fasting in naïve rats, REs, and REs treated with MGE-EV-GABA. REs: Rats with epilepsy; MGE-EV-GABA: GABA loaded into extracellular vesicles (EVs) derived from medial ganglionic eminence cells; For multiple comparisons between the groups, Newman-Keuls post hoc test was used. **p < 0.01; ***p < 0.001; ns: non-significant

Typical performance in this test requires normal perirhinal cortex and the hippocampus of the testing rat. Naïve rats visited novel objects significantly more number times in trial 3 compared to known objects (*p* < 0.01, Fig. 6A), while the differences were nonsignificant in the case of REs (*p* > 0.05). REs treated with MGE-EV-GABA also visited identical times to novel and familiar objects (*p* > 0.05). When the total time spent with each object was compared, naïve rats spent significantly more time with novel objects (*p* < 0.001, Fig. 6A), while REs spent almost equal time with known and novel objects. Interestingly, REs treated with the MGE-EV-GABA spent significantly more time with novel objects than REs (*p* < 0.01). This indicates that MGE-EV-GABA treatment at the given dose partially recovered recognition memory loss in this model.

### MGE-EV-GABA failed to improve the location memory function

An object location test measures spatial learning, and the hippocampal circuitry’s normal functioning is required to complete this task successfully. Naïve rats visited objects in novel places a significantly higher number of times in trial 3 than objects in familiar places (*p* < 0.001, Figure S5). At the same time, the difference was nonsignificant in the case of REs. REs treated with MGE-EV-GABA also visited objects equally in familiar and objects in novel places. Also, when the total time spent with objects in two places was compared, naïve rats spent significantly more time with an object in a novel place (*p* < 0.001, Figure S5), while REs spent almost equal time with familiar and novel place objects. Like REs, REs treated with MGE-EV-GABA also spent a similar time with objects in familiar and novel places. This indicates that MGE-EV-GABA treatment at the given dose failed to recover spatial memory loss seen in this model.

### MGE-EV-GABA improved depressive-like behavior

Epilepsy is well associated with depressive behavior. To understand the efficacy of MGE-EV-GABA treatment in REs, we have performed a modified novelty-suppressed feeding test. In the original NSFT, the test suggested on a new cage. At the same time, in this method, we have completed the test in the home cage to reduce the anxiety associated with REs eating food in an unfamiliar place. Following 24 h of food deprivation, naïve rats reached the food, and the mean latency to the first bite was less than one and a half minutes (Fig. 6B). In this group, 100% of the rats ate food within 5 min of the testing period. In the RE group, the mean latency to the first bite of the food was more than four and a half minutes (Fig. 6B), while > 85% of the tested rats didn’t eat even at 5 min of the testing period. In the MGE-EV-GABA treatment group, the mean latency to the first bite of the food was within 2 min (Fig. 6B), while only 15% of the tested rats didn’t eat at 5 min of the testing period. This data demonstrates that MGE-EV-GABA treatment in REs significantly improved their motivational level, almost equal to naïve rats.

### Exogenously delivered GABA communicates with presynaptic vesicles

**Fig. 7:**
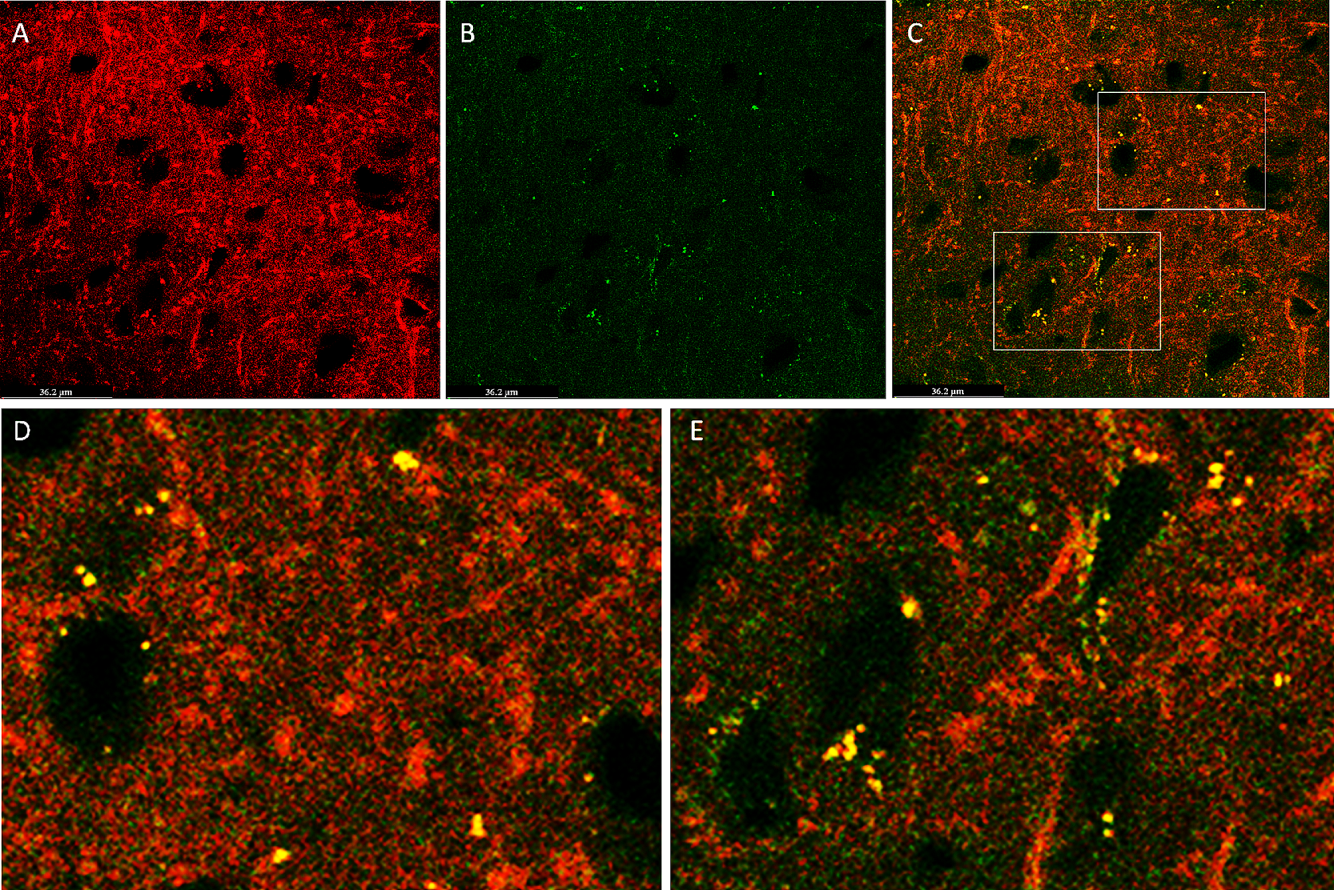
Dual immunostaining of synaptophysin and GABA in the hippocampal dentate hilus of the REs treated with GABA-loaded EVs. Confocal imaging of 0.7 μm Z- section at 630 magnification confirms the co-localization of synaptophysin with GABA in the dentate hilus region. (A) Positive areas for the synaptophysin (red). (B) GABA tagged with o-phthaldialdehyde (green). (C) Co-localization of synaptophysin with GABA (orange). D and E. Inserts from Fig. 7C demonstrating co-localization of synaptophysin and GABA in different areas

To track the exogenously loaded GABA in the brain, rats were intranasally administered with MGE-EVs encapsulated with OPA-conjugated GABA. Dual immunostaining identified synaptophysin (Fig. 7A) and exogenous GABA (Fig. 7B) in the hippocampal dentate hilus of RE rats. Confocal imaging of 0.7 μm Z- section at 630 magnification confirms the co-localization of synaptophysin with GABA in the dentate hilus region (Fig. 7C-E). This co-localization indicates communication between exogenously delivered GABA with presynaptic vesicles, possibly resulting in seizure and partial behavioral control.

## Discussion

Multiple studies have demonstrated that treatment that directly increases the GABAergic inhibitory neurotransmission or reduces the activity of excitatory glutamatergic neurons controls epileptic seizures [14–20]. Also, endogenous GABA produced from transplanted interneurons enhanced functional inhibitory networks. However, most of the ASMs in the market act either on voltage-gated sodium channels or calcium channels, indirectly modulate GABA or glutamate levels, or act on multiple targets, leading to various side effects. As dietary GABA cannot cross the BBB [38], we have identified that exogenous GABA intranasally delivered to the brain could cross the BBBs through EVs from GABA-producing neurons and their progenitors and control seizures. Also, the study recognized that seizure control could be best achieved from GABA delivered through EVs containing parent cell-specific cargo. EVs from interneurons and MGE cells encapsulated with GABA significantly reduced the total number of seizures, the number of stage V seizures (the most severe form of seizure), and the total time spent in seizure activity. In contrast, EVs from NSCs encapsulated with GABA demonstrated limited seizure control in similar conditions. Also, exogenic GABA alone and EVs from INs and MGE cells failed to control seizures. Further, a detailed behavioral analysis of rats treated with MGE-EV-GABA improved depressive behavior while partially improving object-based memory functions. Co-localization studies confirmed the presence of exogenous GABA with presynaptic vesicles in the hippocampus, suggesting that exogenic GABA could modulate the brain with epilepsy.

Extracellular vesicles, with their nano size, are gaining tremendous interest in pharmacology for treating various diseases and drug delivery [39–41]. EVs are considered next-generation drug delivery systems as they can cross BBBs and reach neurons and glial cells [27, 28, 41]. EVs can deliver hydrophilic and hydrophobic drugs [42–43]. As GABA is a small molecule and acts as a zwitterion and cationic in solution, it is suitable for simple passive encapsulation [44]. We have performed 2 experiments to ensure exogenic GABA enters the brain and communicates with it. In the first set of experiments, GABA conjugated with OPA was loaded into PKH 26 labelled EVs. Intranasal administration of these EVs into the naïve rats and the identification of dual immunostained images in the brain confirm that GABA-loaded EVs could reach the brain. In the second set of experiments, GABA conjugated with OPA was loaded into EVs. After 6 h of intranasal administration into rats with epielpsy, confocal imaging confirms the co-localization of synaptophysin with GABA in the dentate hilus region, indicating communication between exogenously delivered GABA with presynaptic vesicles, possibly resulting in seizure and partial behavioral control.

In humans, the half-life of GABA in the blood is 5 h [45]. However, in the human brain, the half-life of GABA is unknown. A single dose of EV-encapsulated GABA demonstrated its effects for at least 10 h in this study, which is a direct indication of its prolonged action, which is at least equivalent to a single dose of a drug per day. Gabapentin, an analogue of GABA used in epilepsy treatment, increased GABA levels in the brain by 57% at 2.5 h following oral administration [46]. Gabapentin is well tolerated but is required in high doses (900–1800 mg/day as a maintenance dose). The bioavailability of gabapentin is 27–60% and is inversely proportional to dosage, while increasing doses and long-term uses are known to cause multiple side effects. Even for exogenic GABA delivery through EVs, side effects, including toxicity, need to be studied in detail. Specifically, changes in GABAergic receptors (internalization or downregulation) induced by chronic exposure to GABA need to be evaluated.

Identifying a suitable source of EVs for specific drug delivery is essential, as the cargo of the delivered EVs shouldn’t alter the recipient cell functionality other than the intended purpose. This is a necessary issue as EV-transferred components could be functional in the recipient cells and change their function [47]. In this study, we intend to deliver GABA to the hippocampus, which contains 10–15% of GABAergic interneurons among total neurons that control diverse cellular and circuit functions through innumerable synapses. Their reduced numbers are well-established in epilepsy [48, 49]. Thus, EVs from GABAergic interneurons are the best bet for encapsulating GABA and delivering it to the hippocampus. With directed differentiation, NSCs, MGE cells, and GABAergic interneurons could be reproducibly generated from human pluripotent stem cells [50, 51]. However, the expense of generating interneurons is high compared to MGEs. Also, the interneuron generation requires 45 days of culture, while the MGE generation requires 25 days [50]. Indeed, co-localization of the MGE-EV delivered exogenous GABA with the presynaptic vesicle in the hippocampus and seizure control confirms the capability of the MGE-EVs similar to IN-EV-GABA. Further, consistent reduction of seizures for a week in IN-EV-GABA and MGE-EV-GABA but not with NSC-EV-GABA demonstrates the essential roles of EV cargo in seizure control along with GABA. Since the size, shape, morphology, and marker expressions were comparable between EVs derived from NSCs, MGE cells, and interneurons, likely, distinct cell type-specific EV cargo components such as mRNAs, miRNAs, lncRNAs, proteins and/or lipids, etc. could essentially contribute for seizure control through synaptic modulation. A recent study demonstrated improvement of GABA synaptic function in Huntington’s disease neurons following treatment with EVs from fibroblasts through miRNA modulation [52].

Cognitive and mood impairments are well associated with temporal lobe epilepsy and are linked through different mechanisms [53]. Also, ASMs cause multiple side effects in the cognitive domain [8–9]. However, MGE-EV-GABA treatment in the study demonstrated significant improvements in cognitive behavior compared to untreated rats. Improvements in behavioral function may be due to synchronization in excitatory and inhibitory neuronal functions [54] induced by exogenously delivered GABA and cell-specific EV cargo. We believe that continuous treatment could further control seizures and modulate behavioral impairments. Dose and time-dependent studies are required to address these issues further.

As MGE-EV encapsulated exogenous GABA could control seizures and behavior in epilepsy, it needs to be tested for its efficacy against pharmaco-resistant epilepsy, an essential clinical target of such novel approaches as none of the medications in the last 25 years could reduce the incidence of pharmaco-resistant epilepsy [4, 55–56]. Also, as multiple clinical trials are being conducted with EVs [57] and EVs can be cryopreserved without damaging their potential [58], we believe cell-specific EVs hold great promise in the drug delivery field for multiple clinical applications, including pharmaco-resistant epilepsy.

Limitations of the study include the fact that detailed pharmacokinetics and pharmacodynamics of EV-encapsulated GABA in the brain have not been studied. We used a single concentration of GABA daily, as it is the maximum dose we could load. Increasing loading efficiency through EV bioengineering could help load higher concentrations of GABA [59], while dose-dependent studies could help to understand the specific dose required to control seizures. As clinical applications of EVs are gaining tremendous interest, long-term safety studies with these EVs are needed [60]. Another major limitation is that we have used a single iPSC line for the study. Also, rather than iPSC-derived cells, EVs from actual human NSCs, MGE cells and interneurons would have improved the quality of the study. Using a 100% pure population of NSCs, MGE cells, interneurons and derived EVs and their robust molecular characterization could further strengthen the findings. Also, evaluation of protein, mRNA, miRNA and lncRNA composition of EVs cargo of NSCs, MGE cells and GABAergic interneurons through proteomic and transcriptomic analysis could help to trace cargo specificity for seizure control along with GABA for treating epilepsy.

## Conclusions

This study identified three essential findings. (1) Exogenic GABA could be delivered to the brain through brain cell-derived EVs. (2) Exogenic GABA with EV cargo could modulate seizures in temporal lobe epilepsy. (3) The cellular origin of EVs plays a vital role in seizure control with exogenic GABA. The limitations addressed in the study need to be addressed with multiple additional experiments to consider EV-encapsulated GABA as the finest stem cell-derived biotherapeutic agent for treating TLE.

### Electronic supplementary material

Below is the link to the electronic supplementary material.

Supplementary Material 1

## Declarations

Title of the approved project: “Exosomes from human pluripotent stem cells derived GABAergic interneuron progenitors: Next generation vehicles for treating GABA disorders?”

Name of the institutional approval committee or unit: Institutional Animal Ethics Committee, Kasturba Medical College, Manipal Academy of Higher Education, Manipal.

Approval number: IAEC/KMC/39/2018.

Date of approval: May 26, 2018.

### Availability of supporting data

All data generated or analyzed during this study are included in this published article.

## References

[1] Devinsky O. Diagnosis and treatment of temporal lobe epilepsy. Rev Neurol Dis. 2004 Winter;1(1):2–9. 16397445

[2] Lewis DV (2005). Losing neurons: selective vulnerability and mesial temporal sclerosis. Epilepsia.

[3] Kwan P, Arzimanoglou A, Berg AT, Brodie MJ, Allen Hauser W, Mathern G, Moshé SL, Perucca E, Wiebe S, French J (2010). Definition of drug resistant epilepsy: consensus proposal by the ad hoc Task Force of the ILAE Commission on therapeutic strategies. Epilepsia.

[4] Golyala A, Kwan P (2017). Drug development for refractory epilepsy: the past 25 years and beyond. Seizure.

[5] Rocha LL, Cavalheiro EA, Lazarowski A, Rocha LL, Lazarowski A, Cavalheiro EA (2023). Why Study Drug-Resistant Epilepsy?. Pharmacoresistance in Epilepsy.

[6] Brodie MJ (2017). Sodium Channel Blockers in the treatment of Epilepsy. CNS Drugs.

[7] Sills GJ, Rogawski MA (2020). Mechanisms of action of currently used anti-seizure drugs. Neuropharmacology.

[8] Chen B, Choi H, Hirsch LJ, Katz A, Legge A, Buchsbaum R, Detyniecki K (2017). Psychiatric and behavioral side effects of antiepileptic drugs in adults with epilepsy. Epilepsy Behav.

[9] de Kinderen RJ, Evers SM, Rinkens R, Postulart D, Vader CI, Majoie MH, Aldenkamp AP (2014). Side-effects of antiepileptic drugs: the economic burden. Seizure.

[10] Righes Marafiga J, Vendramin Pasquetti M, Calcagnotto ME (2021). GABAergic interneurons in epilepsy: more than a simple change in inhibition. Epilepsy Behav.

[11] Scharfman HE (2007). The neurobiology of epilepsy. Curr Neurol Neurosci Rep.

[12] Zhu Q, Mishra A, Park JS, Liu D, Le DT, Gonzalez SZ, Anderson-Crannage M, Park JM, Park GH, Tarbay L, Daneshvar K, Brandenburg M, Signoretti C, Zinski A, Gardner EJ, Zheng KL, Abani CP, Hu C, Beaudreault CP, Zhang XL, Stanton PK, Cho JH, Velíšek L, Velíšková J, Javed S, Leonard CS, Kim HY, Chung S (2023). Human cortical interneurons optimized for grafting specifically integrate, abort seizures, and display prolonged efficacy without over-inhibition. Neuron.

[13] Huberfeld G, Menendez de la Prida L, Pallud J, Cohen I, Le Van Quyen M, Adam C, Clemenceau S, Baulac M, Miles R (2011). Glutamatergic pre-ictal discharges emerge at the transition to seizure in human epilepsy. Nat Neurosci.

[14] Zhou QG, Nemes AD, Lee D, Ro EJ, Zhang J, Nowacki AS, Dymecki SM, Najm IM, Suh H (2019). Chemogenetic silencing of hippocampal neurons suppresses epileptic neural circuits. J Clin Invest.

[15] Hunt RF, Girskis KM, Rubenstein JL, Alvarez-Buylla A, Baraban SC (2013). GABA progenitors grafted into the adult epileptic brain control seizures and abnormal behavior. Nat Neurosci.

[16] Cunningham M, Cho JH, Leung A, Savvidis G, Ahn S, Moon M, Lee PK, Han JJ, Azimi N, Kim KS, Bolshakov VY, Chung S (2014). hPSC-derived maturing GABAergic interneurons ameliorate seizures and abnormal behavior in epileptic mice. Cell Stem Cell.

[17] Henderson KW, Gupta J, Tagliatela S, Litvina E, Zheng X, Van Zandt MA, Woods N, Grund E, Lin D, Royston S, Yanagawa Y, Aaron GB, Naegele JR (2014). Long-term seizure suppression and optogenetic analyses of synaptic connectivity in epileptic mice with hippocampal grafts of GABAergic interneurons. J Neurosci.

[18] Upadhya D, Hattiangady B, Castro OW, Shuai B, Kodali M, Attaluri S, Bates A, Dong Y, Zhang SC, Prockop DJ, Shetty AK (2019). Human induced pluripotent stem cell-derived MGE cell grafting after status epilepticus attenuates chronic epilepsy and comorbidities via synaptic integration. Proc Natl Acad Sci U S A.

[19] Upadhya D, Attaluri S, Liu Y, Hattiangady B, Castro OW, Shuai B, Dong Y, Zhang SC, Shetty AK (2022). Grafted hPSC-derived GABA-ergic interneurons regulate seizures and specific cognitive function in temporal lobe epilepsy. NPJ Regen Med.

[20] Arshad MN, Pinto A, van Praag H, Naegele JR (2023). Altered connectomes of adult-born granule cells following engraftment of GABAergic progenitors in the mouse hippocampus. Prog Neurobiol.

[21] Hepsomali P, Groeger JA, Nishihira J, Scholey A (2020). Effects of oral Gamma-Aminobutyric Acid (GABA) administration on stress and sleep in humans: a systematic review. Front Neurosci.

[22] Tower DB, Roberts E, Baxter CF, van Harreveld A, Wiersma CAG, Adey WR, Killam KF (1960). The administration of gamma-aminobutyric acid to man: systemic effects and anticonvulsant action. Inhibition in the nervous system and Gamma-Aminobutyric Acid.

[23] Kuriyama K, Sze PY (1971). Blood-brain barrier to H3-gamma-aminobutyric acid in normal and amino oxyacetic acid-treated animals. Neuropharmacology.

[24] Kakee A, Takanaga H, Terasaki T, Naito M, Tsuruo T, Sugiyama Y (2001). Efflux of a suppressive neurotransmitter, GABA, across the blood–brain barrier. J Neurochem.

[25] Yurtdaş Kırımlıoğlu G, Menceloğlu Y, Erol K, Yazan Y (2016). In vitro/in vivo evaluation of gamma-aminobutyric acid-loadedN,N-dimethylacrylamide-based pegylated polymeric nanoparticles for brain delivery to treat epilepsy. J Microencapsul.

[26] Fontes MAP, Vaz GC, Cardoso TZD, de Oliveira MF, Campagnole-Santos MJ, Dos Santos RAS, Sharma NM, Patel KP, Frézard F (2018). GABA-containing liposomes: neuroscience applications and translational perspectives for targeting neurological diseases. Nanomedicine.

[27] Long Q, Upadhya D, Hattiangady B, Kim DK, An SY, Shuai B, Prockop DJ, Shetty AK (2017). Intranasal MSC-derived A1-exosomes ease inflammation, and prevent abnormal neurogenesis and memory dysfunction after status epilepticus. Proc Natl Acad Sci U S A.

[28] Banks WA, Sharma P, Bullock KM, Hansen KM, Ludwig N, Whiteside TL (2020). Transport of Extracellular vesicles across the blood-brain barrier: Brain Pharmacokinetics and effects of inflammation. Int J Mol Sci.

[29] Hurwitz SN, Conlon MM, Rider MA, Brownstein NC, Meckes DG (2016). Nanoparticle analysis sheds budding insights into genetic drivers of extracellular vesicle biogenesis. J Extracell Vesicles.

[30] Rider MA, Hurwitz SN, Meckes DG (2016). ExtraPEG: a polyethylene glycol-based Method for Enrichment of Extracellular vesicles. Sci Rep.

[31] García-Romero N, Madurga R, Rackov G, Palacín-Aliana I, Núñez-Torres R, Asensi-Puig A (2019). Polyethylene glycol improves current methods for circulating extracellular vesicle-derived DNA isolation. J Transl Med.

[32] Somiya M, Kuroda S (2021). Reporter gene assay for membrane fusion of extracellular vesicles. J Extracell Vesicles.

[33] Roy S, Kashyap NN, Anchan AS, Punja D, Jasti DB, Upadhya D (2023). Urinary extracellular vesicle dynamics in Parkinson’s disease patients with urinary dysfunction. Front Neurol.

[34] Welsh JA, Goberdhan DCI, O’Driscoll L, Buzas EI, Blenkiron C, Bussolati B, Cai H, Di Vizio D, Driedonks TAP, Erdbrügger U, Falcon-Perez JM, Fu QL, Hill AF, Lenassi M, Lim SK, Mahoney MG, Mohanty S, Möller A, Nieuwland R, Ochiya T, Sahoo S, Torrecilhas AC, Zheng L, Zijlstra A, Abuelreich S, Bagabas R, Bergese P, Bridges EM, Brucale M, Burger D, Carney RP, Cocucci E, Crescitelli R, Hanser E, Harris AL, Haughey NJ, Hendrix A, Ivanov AR, Jovanovic-Talisman T, Kruh-Garcia NA, Ku’ulei-Lyn Faustino, Kyburz V, Lässer D, Lennon C, Lötvall KM, Maddox J, Martens-Uzunova AL, Mizenko ES, Newman RR, Ridolfi LA, Rohde A, Rojalin E, Rowland T, Saftics A, Sandau A, Saugstad US, Shekari JA, Swift F, Ter-Ovanesyan S, Tosar D, Useckaite JP, Valle Z, Varga F, van der Pol Z, van Herwijnen E, Wauben MJC, Wehman MHM, Williams AM, Zendrini S, Zimmerman A. AJ; MISEV Consortium; Théry C, Witwer KW. Minimal information for studies of extracellular vesicles (MISEV2023): From basic to advanced approaches. J Extracell Vesicles. 2024;13(2):e12404. 10.1002/jev2.12404 PMC10850029 38326288

[35] Haney MJ, Klyachko NL, Zhao Y, Gupta R, Plotnikova EG, He Z, Patel T, Piroyan A, Sokolsky M, Kabanov AV, Batrakova EV (2015). Exosomes as drug delivery vehicles for Parkinson’s disease therapy. J Control Release.

[36] Upadhya D, Kodali M, Gitai D, Castro OW, Zanirati G, Upadhya R, Attaluri S, Mitra E, Shuai B, Hattiangady B, Shetty AK (2019). A model of chronic temporal lobe Epilepsy presenting constantly rhythmic and robust spontaneous Seizures, co-morbidities and hippocampal neuropathology. Aging Dis.

[37] Vogel-Ciernia A, Wood MA (2014). Examining object location and object recognition memory in mice. Curr Protoc Neurosci.

[38] Boonstra E, de Kleijn R, Colzato LS, Alkemade A, Forstmann BU, Nieuwenhuis S (2015). Neurotransmitters as food supplements: the effects of GABA on brain and behavior. Front Psychol.

[39] Zhang Y, Belaid M, Luo X, Daci A, Limani R, Mantaj J, Zilbauer M, Nayak K, Vllasaliu D (2023). Probing milk extracellular vesicles for intestinal delivery of RNA therapies. J Nanobiotechnol.

[40] Upadhya D, Shetty AK (2019). Extracellular vesicles as therapeutics for Brain Injury and Disease. Curr Pharm Des.

[41] Saint-Pol J, Gosselet F, Duban-Deweer S, Pottiez G, Karamanos Y (2020). Targeting and crossing the blood-brain barrier with Extracellular vesicles. Cells.

[42] Hettich BF, Bader JJ, Leroux JC (2022). Encapsulation of Hydrophilic compounds in small extracellular vesicles: loading capacity and impact on vesicle functions. Adv Healthc Mater.

[43] Reddy SK, Ballal AR, Shailaja S, Seetharam RN, Raghu CH, Sankhe R, Pai K, Tender T, Mathew M, Aroor A, Shetty AK, Adiga S, Devi V, Muttigi MS, Upadhya D (2023). Small extracellular vesicle-loaded bevacizumab reduces the frequency of intravitreal injection required for diabetic retinopathy. Theranostics.

[44] Fabbiani FP, Buth G, Levendis DC, Cruz-Cabeza AJ (2014). Pharmaceutical hydrates under ambient conditions from high-pressure seeds: a case study of GABA monohydrate. Chem Commun.

[45] Li J, Zhang Z, Liu X, Wang Y, Mao F, Mao J, Lu X, Jiang D, Wan Y, Lv JY, Cao G, Zhang J, Zhao N, Atkinson M, Greiner DL, Prud’homme GJ, Jiao Z, Li Y, Wang Q (2015). Study of GABA in healthy volunteers: pharmacokinetics and Pharmacodynamics. Front Pharmacol.

[46] Cai K, Nanga RP, Lamprou L, Schinstine C, Elliott M, Hariharan H, Reddy R, Epperson CN (2012). The impact of gabapentin administration on brain GABA and glutamate concentrations: a 7T ¹H-MRS study. Neuropsychopharmacology.

[47] Chen J, Ma S, Luo B, Hao H, Li Y, Yang H, Zhu F, Zhang P, Niu R, Pan P (2023). Human umbilical cord mesenchymal stromal cell small extracellular vesicle transfer of microRNA-223-3p to lung epithelial cells attenuates inflammation in acute lung injury in mice. J Nanobiotechnol.

[48] Pelkey KA, Chittajallu R, Craig MT, Tricoire L, Wester JC, McBain CJ (2017). Hippocampal GABAergic inhibitory interneurons. Physiol Rev.

[49] Shetty AK, Upadhya D (2016). GABA-ergic cell therapy for epilepsy: advances, limitations and challenges. Neurosci Biobehav Rev.

[50] Liu Y, Liu H, Sauvey C, Yao L, Zarnowska ED, Zhang SC (2013). Directed differentiation of forebrain GABA interneurons from human pluripotent stem cells. Nat Protoc.

[51] Upadhya D, Hattiangady B, Shetty GA, Zanirati G, Kodali M, Shetty AK. Neural Stem Cell or Human Induced Pluripotent Stem Cell-Derived GABA-ergic Progenitor Cell Grafting in an Animal Model of Chronic Temporal Lobe Epilepsy. Curr Protoc Stem Cell Biol. 2016; 38:2D.7.1-2D.7.47. 10.1002/cpsc.9 PMC5313261 27532817

[52] Beatriz M, Rodrigues RJ, Vilaça R, Egas C, Pinheiro PS, Daley GQ, Schlaeger TM, Raimundo N, Rego AC, Lopes C (2023). Extracellular vesicles improve GABAergic transmission in Huntington’s disease iPSC-derived neurons. Theranostics.

[53] Khalife MR, Scott RC, Hernan AE (2022). Mechanisms for cognitive impairment in Epilepsy: moving Beyond seizures. Front Neurol.

[54] Lenck-Santini PP. RC Scott 2015 Mechanisms responsible for cognitive impairment in epilepsy. Cold Spring Harb Perspect Med 5 pii: a022772. 10.1101/cshperspect.a022772 PMC4588128 26337111

[55] Orozco-Suárez S, Feria-Romero IA, Ureña-Guerrero ME, Rocha LL, Alonso-Vanegas MA. GABAergic Neurotransmission Abnormalities in Pharmacoresistant Epilepsy: Experimental and Human Studies. Pharmacoresistance in epilepsy: from genes and molecules to promising therapies. Second edition. Springer Nature, 2023, 335–369.

[56] Servilha-Menezes G, Garcia-Cairasco N (2022). A complex systems view on the current hypotheses of epilepsy pharmacoresistance. Epilepsia Open.

[57] Duong A, Parmar G, Kirkham AM, Burger D, Allan DS (2023). Registered clinical trials investigating treatment with cell-derived extracellular vesicles: a scoping review. Cytotherapy.

[58] Guarro M, Suñer F, Lecina M, Borrós S, Fornaguera C (2022). Efficient extracellular vesicles freeze-dry method for direct formulations preparation and use. Colloids Surf B Biointerfaces.

[59] Jayasinghe MK, Pirisinu M, Yang Y, Peng B, Pham TT, Lee CY, Tan M, Vu LT, Dang XTT, Pham TC, Chen H, Leung AYH, Cho WC, Shi J, Le MTN (2022). Surface-engineered extracellular vesicles for targeted delivery of therapeutic RNAs and peptides for cancer therapy. Theranostics.

[60] Cheng K (2023). Raghu Kalluri. Guidelines for clinical translation and commercialization of extracellular vesicles and exosomes based therapeutics. Extracell Vesicles.

