# Supplementary material for: Cell-specific extracellular vesicle-encapsulated exogenous GABA controls seizures in epilepsy
Source: Stem Cell Res Ther. 2024 Apr 19;15:108. doi: 10.1186/s13287-024-03721-4 (PMC11027552; doi:10.1186/s13287-024-03721-4)
Supplement: Supplementary file 1 — Supplementary Material 1 [file 13287_2024_3721_MOESM1_ESM.docx]

Supplementary figures


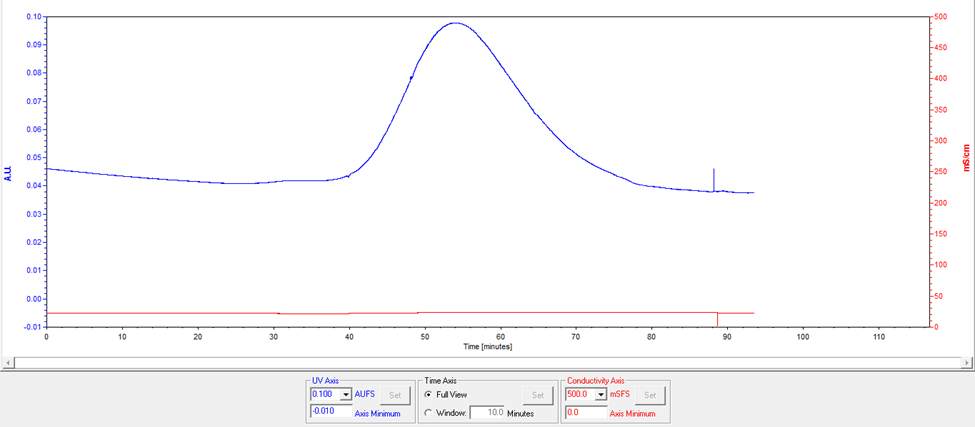


Figure S1: Conjugation of GABA with o-phthaldialdehyde (OPA). GABA was conjugated with o-phthaldialdehyde (OPA) for tracking in the rat brain. During elution, the fluorescent GABA-OPA peak was observed after 40 minutes, and the fraction was collected and purified.

Original western blot images (For Figure 3C, for NSC-EVs)


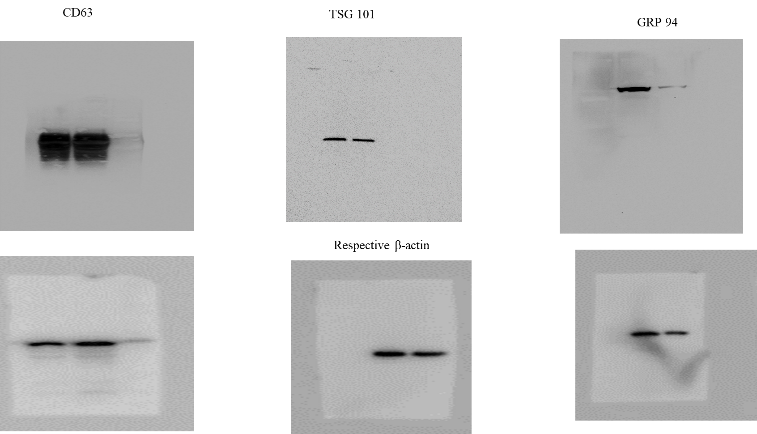


Original western blot images (For Figure 3C, for MGE-EVs)


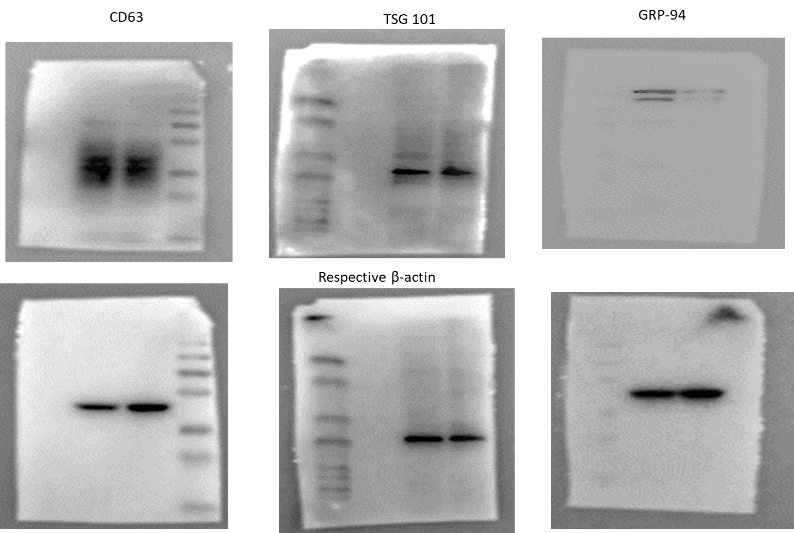


Original western blot images (For Figure 3C, for IN-EVs)


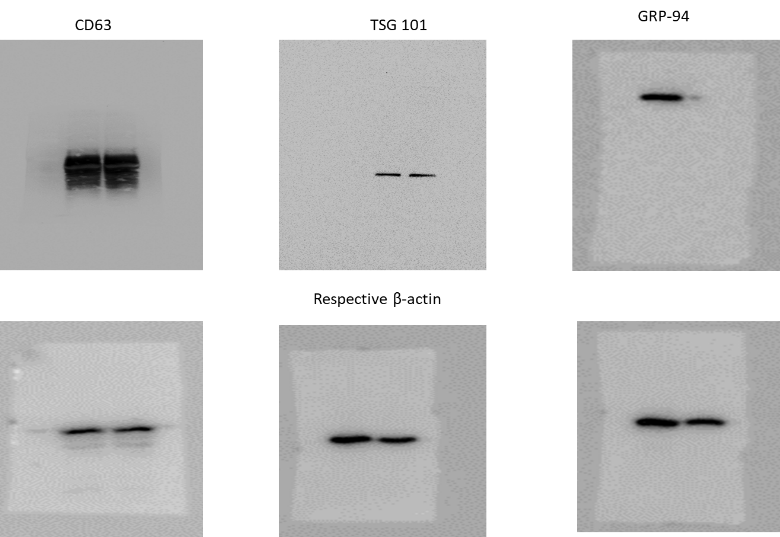


Figure S2: Western blot original images for figure 3.


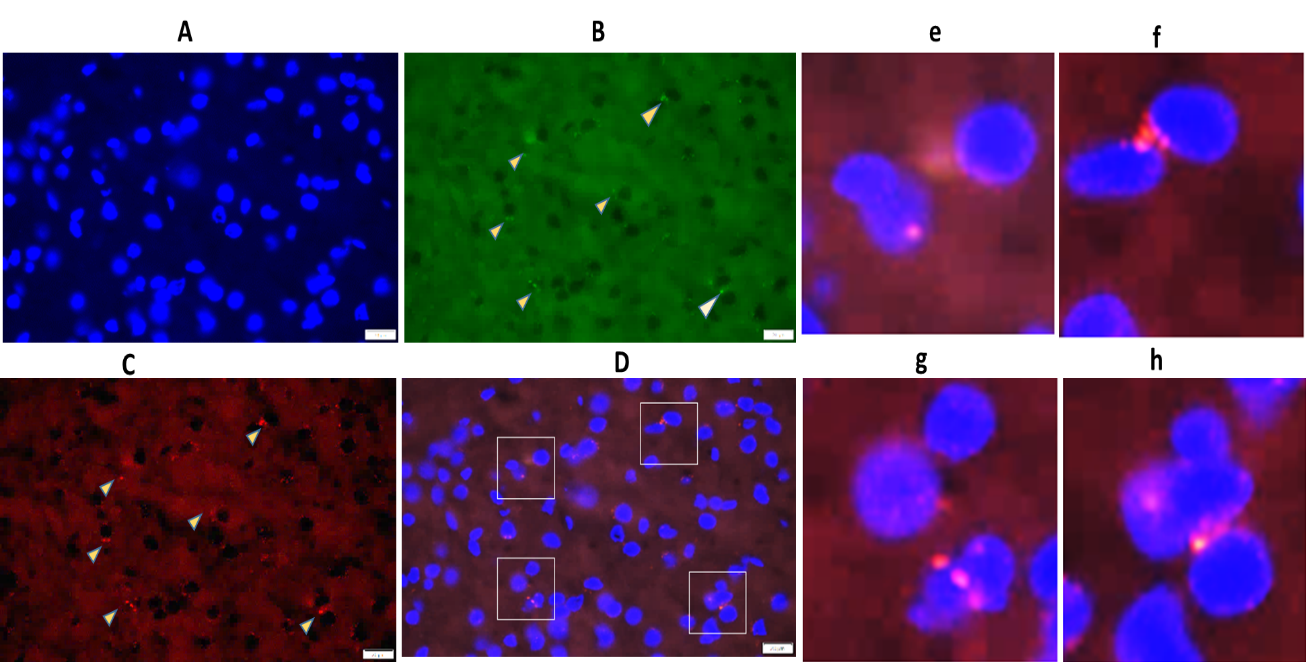


Figure S3: Tracking GABA loaded EVs in the brain. Following intranasal administration of GABA-OPA-loaded EVs into rats, immunofluorescence studies of the brain revealed the presence of GABA-OPA (green) and EV-PKH 26 (red) in the hippocampus. A: DAPI; B GABA-OPA; C: EV-PKH 26; D: Merged image. e-f, g and h are enlarged inserts from D. Arrowheads were used for demonstrating positive staining.

**
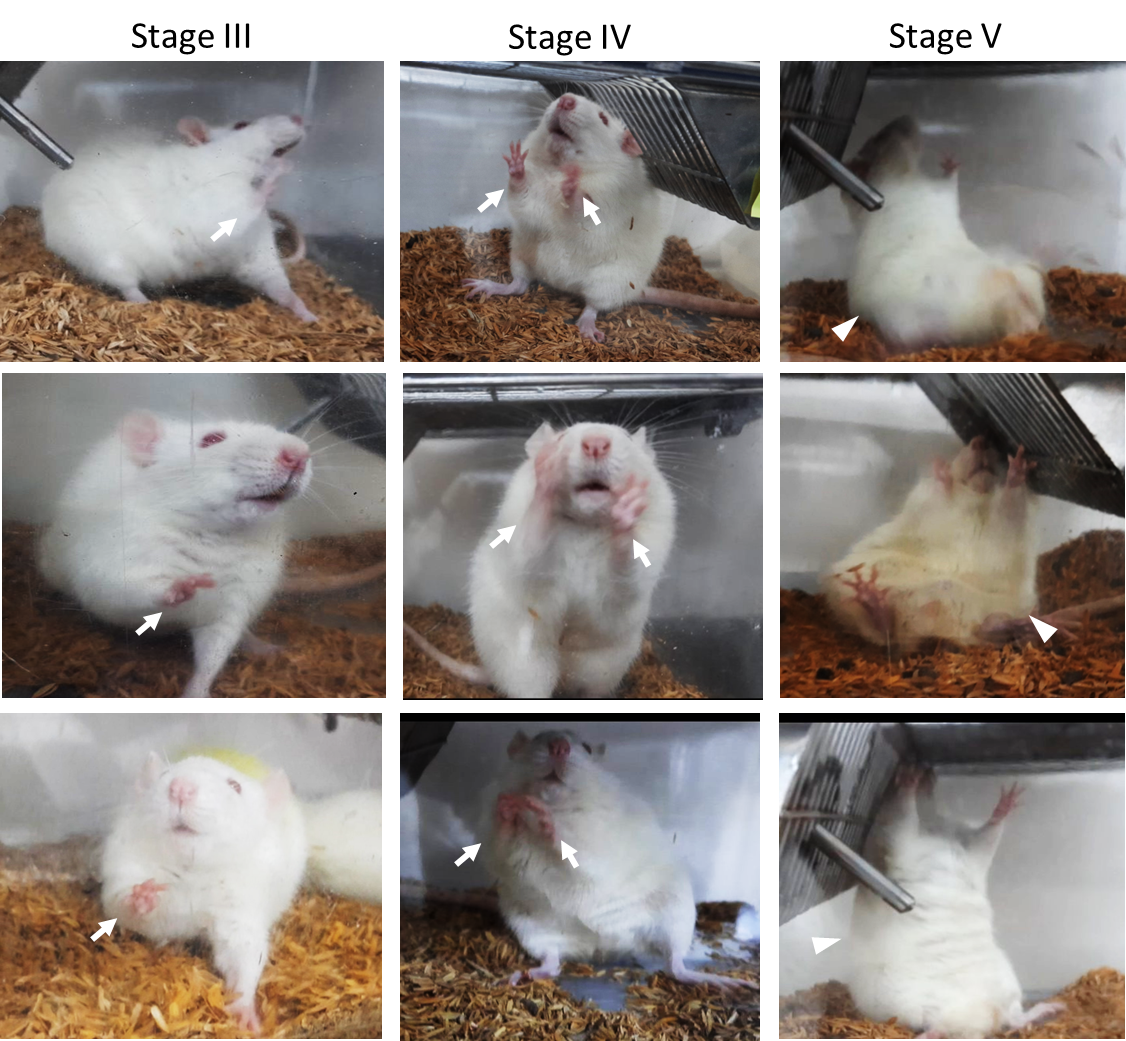
**

Figure S4: Epilepsy development in rats: scoring of seizures based on Racine scale. The left panel demonstrates stage III seizures displaying unilateral forelimb clonus as marked by arrows. The middle panel demonstrates stage IV seizures displaying bilateral forelimb clonus and rearing, as marked by arrows, while the right panel demonstrates stage V seizures displaying bilateral forelimb clonus with rearing and falling, as demonstrated by arrowheads.


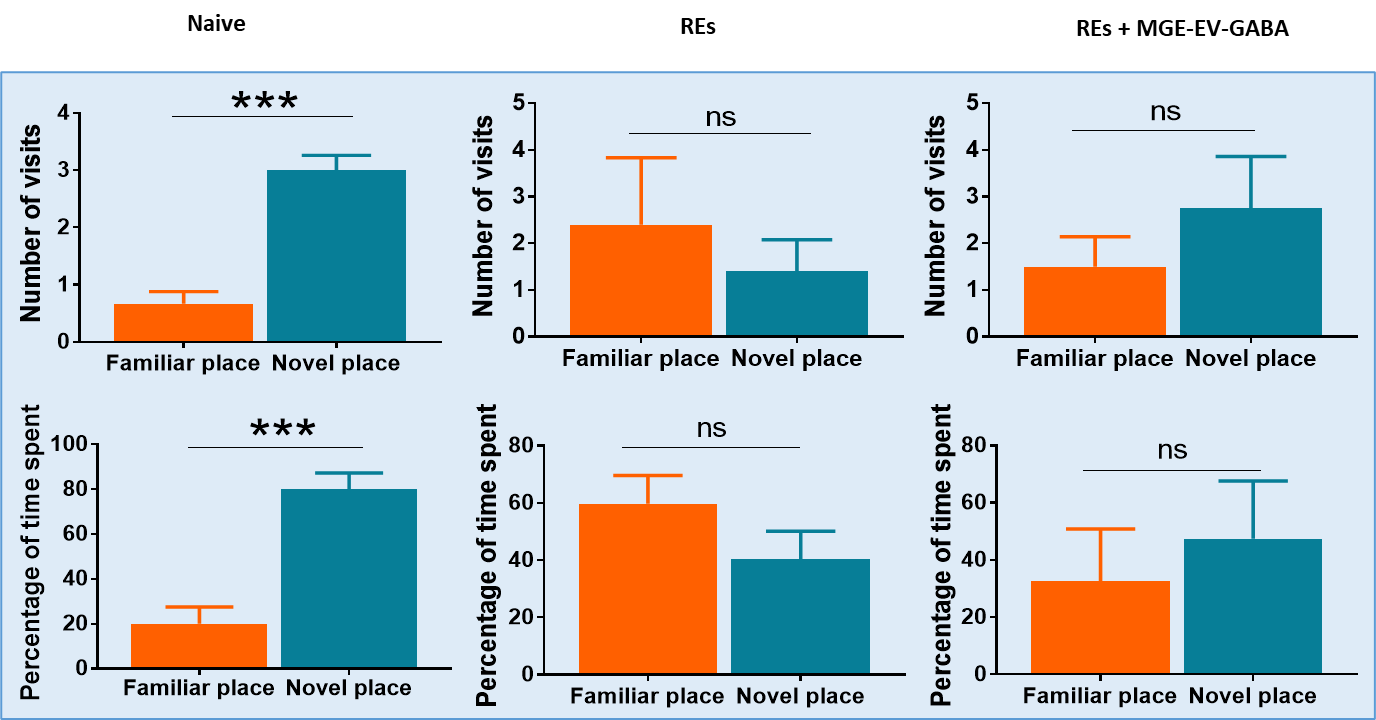


Figure S5: The upper panel demonstrates the number of visits to novel and known objects in trial 3 from the group of naïve rats, REs and REs treated with MGE-EV-GABA. The lower panel demonstrates the total time spent with each object in trial 3 from the group belonging to naïve rats, REs and REs treated with MGE-EV-GABA.
